# Supplementary material for: Resolving the full spectrum of human genome variation using Linked-Reads
Source: Genome Res. 2019 Apr;29(4):635–45. doi: 10.1101/gr.234443.118 (PMC6442396; doi:10.1101/gr.234443.118)
Supplement: Supplemental Material [file supp_gr.234443.118_Supplemental_longranger-2.2.zip › longranger-2.2.tar.bz2!/lr2.2-public/lib/stan/cmdstan/stan/doc/api/html/agrad_2rev_2functions_2fdim_8hpp_source.html]

Stan: src/stan/agrad/rev/functions/fdim.hpp Source File


|  |  |
| --- | --- |
|  | Stan  2.5.0  probability, sampling & optimization |


- Main Page
- Related Pages
- Namespaces
- Classes
- Files

- File List
- File Members

All Classes Namespaces Files Functions Variables Typedefs Enumerations Enumerator Friends Macros Pages

- src
- stan
- agrad
- rev
- functions

fdim.hpp

Go to the documentation of this file.

1 #ifndef STAN\_\_AGRAD\_\_REV\_\_FUNCTIONS\_\_FDIM\_HPP

2 #define STAN\_\_AGRAD\_\_REV\_\_FUNCTIONS\_\_FDIM\_HPP

3

4 #include <stan/agrad/rev/var.hpp>

5 #include <stan/agrad/rev/internal/vv\_vari.hpp>

6 #include <stan/agrad/rev/internal/vd\_vari.hpp>

7 #include <stan/agrad/rev/internal/dv\_vari.hpp>

8 #include <boost/math/special\_functions/fpclassify.hpp>

9 #include <stan/meta/likely.hpp>

10

11 namespace stan {

12  namespace agrad {

13

14  namespace {

15  class fdim\_vv\_vari : public op\_vv\_vari {

16  public:

17  fdim\_vv\_vari(vari\* avi, vari\* bvi) :

18  op\_vv\_vari(avi->val\_ - bvi->val\_, avi, bvi) {

19  }

20  void chain() {

21  if (unlikely(boost::math::isnan(avi\_->val\_)

22  || boost::math::isnan(bvi\_->val\_))) {

23  avi\_->adj\_ = std::numeric\_limits<double>::quiet\_NaN();

24  bvi\_->adj\_ = std::numeric\_limits<double>::quiet\_NaN();

25  } else {

26  avi\_->adj\_ += adj\_;

27  bvi\_->adj\_ -= adj\_;

28  }

29  }

30  };

31

32  class fdim\_vd\_vari : public op\_vd\_vari {

33  public:

34  fdim\_vd\_vari(vari\* avi, double b) :

35  op\_vd\_vari(avi->val\_ - b, avi,b) {

36  }

37  void chain() {

38  if (unlikely(boost::math::isnan(avi\_->val\_)

39  || boost::math::isnan(bd\_)))

40  avi\_->adj\_ = std::numeric\_limits<double>::quiet\_NaN();

41  else

42  avi\_->adj\_ += adj\_;

43  }

44  };

45

46  class fdim\_dv\_vari : public op\_dv\_vari {

47  public:

48  fdim\_dv\_vari(double a, vari\* bvi) :

49  op\_dv\_vari(a - bvi->val\_, a,bvi) {

50  }

51  void chain() {

52  if (unlikely(boost::math::isnan(bvi\_->val\_)

53  || boost::math::isnan(ad\_)))

54  bvi\_->adj\_ = std::numeric\_limits<double>::quiet\_NaN();

55  else

56  bvi\_->adj\_ -= adj\_;

57  }

58  };

59  }

60

112  inline var fdim(const stan::agrad::var& a,

113  const stan::agrad::var& b) {

114  if (!(a.vi\_->val\_ <= b.vi\_->val\_))

115  return var(new fdim\_vv\_vari(a.vi\_,b.vi\_));

116  else

117  return var(new vari(0.0));

118  }

119

137  inline var fdim(const double& a,

138  const stan::agrad::var& b) {

139  return a <= b.vi\_->val\_

140  ? var(new vari(0.0))

141  : var(new fdim\_dv\_vari(a,b.vi\_));

142  }

143

160  inline var fdim(const stan::agrad::var& a,

161  const double& b) {

162  return a.vi\_->val\_ <= b

163  ? var(new vari(0.0))

164  : var(new fdim\_vd\_vari(a.vi\_,b));

165  }

166

167  }

168 }

169 #endif

boost::math::isnan

bool isnan(const stan::agrad::var &v)

Checks if the given number is NaN.

**Definition:** boost\_fpclassify.hpp:69

dv\_vari.hpp

stan::agrad::vari::val\_

const double val\_

The value of this variable.

**Definition:** vari.hpp:37

vd\_vari.hpp

stan::agrad::var::vi\_

vari \* vi\_

Pointer to the implementation of this variable.

**Definition:** var.hpp:40

unlikely

#define unlikely(x)

**Definition:** likely.hpp:9

stan::agrad::vari

The variable implementation base class.

**Definition:** vari.hpp:28

var.hpp

likely.hpp

stan::agrad::var

Independent (input) and dependent (output) variables for gradients.

**Definition:** var.hpp:27

vv\_vari.hpp

stan::agrad::fdim

fvar< T > fdim(const fvar< T > &x1, const fvar< T > &x2)

**Definition:** fdim.hpp:15


---

[ Stan Home Page ]

*© 2011–2014,
Stan Development Team.*
